# Compensating the Meniscus Effect in Phase Contrast Microscopy Using an LCD for Adaptive Condenser Annulus Shifting

**DOI:** 10.1002/jemt.24808

**Published:** 2025-01-17

**Authors:** Florian Nienhaus, Finn Burkhardt, Niels König, Robert H. Schmitt

**Affiliations:** ^1^ Fraunhofer Institute for Production Technology IPT Aachen Germany; ^2^ WZL, RWTH Aachen University Aachen Germany

**Keywords:** image analysis, LCD condenser annulus, meniscus effect compensation, microtiter plates (MTPs), phase contrast microscopy

## Abstract

The meniscus effect in cell culture vessels limits the observable areas with phase contrast microscopy. For meniscus effect compensation in microtiter plates (MTPs), we present a method using an LCD to replace the fixed condenser annulus, which enables adaptive annulus shifting based on image analysis. This approach led to an increase in phase contrast area by a factor of 8.3. Utilizing a standard phase contrast microscope, we substituted the static condenser annulus with a transparent LCD that displays an adaptive annulus, which can be repositioned to counteract meniscus‐induced refraction across an entire MTP‐24 well. We developed image analysis using Bertrand lens images to determine the misalignment between annulus center and phase ring, enabling the calculation of the required annulus shift. Experiments demonstrate the effectiveness of this image analysis technique. The detected shift was translated into new LCD settings through a linear regression model to ensure proper alignment for the following image. We proved that an algorithm based on background brightness yields a reliable metric for assessing phase contrast conditions within well‐plates. The proposed approach substantially increased the phase contrast area in 24‐well MTPs at 10× magnification from 5.0% with conventional microscopy to 41.9%, thereby restoring phase contrast conditions throughout the well, except near the edges.

## Graphical abstract

We present a method to compensate for the meniscus effect in phase contrast microscopy by using an LCD instead of a fixed condenser annulus. This approach enables adaptive annulus shifting based on image analysis, resulting in an increase in the phase contrast area by a factor of 8.3.

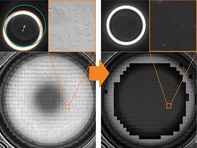

**Summary**

- A novel method using a transmissive LCD as an adaptive condenser annulus in phase contrast microscopy is introduced, effectively counteracting the meniscus effect.
- An innovative technique to quantify the phase contrast area in well‐plates was developed.
- A robust process accurately determines the condenser annulus shift in MTPs through image analysis.
- The approach significantly increased the phase contrast area to 41.9% in MTP24 wells, compared with only 5.0% with conventional methods.

## 1 Introduction

With the increasing degree of laboratory automation, quality measurement methods must evolve to satisfy more stringent requirements, such as enhanced measurement speed and the assessment of 100% of all samples, as opposed to relying on random sampling (Biermann et al. 2021). Phase contrast microscopy is a widely employed technique for cell quality control in laboratories. Since its invention in the 1930s by Frits Zernike, for which he was awarded the Nobel Prize in Physics, it has become indispensable in various scientific applications (Zernike 1942; Bennet et al. 1952). It enhances contrast in transparent specimens without the need for staining (Yin, Kanade, and Chen 2012). By being non‐invasive, it is a popular alternative to fluorescence microscopy (Jaccard et al. 2014; Davis et al. 2007).

Applications such as drug discovery require complete control over specimens, ideally scanning 100% of the area (Lin et al. 2020; Blay et al. 2020). This is crucial for biological samples in rare disease research and personalized medicine (Ochs et al. 2022; Hopkins, Keane, and Balaskas 2020; Guiot et al. 2022). These samples are typically cultivated in microtiter plates (MTPs), which consist of cylindrical wells filled with liquid.

Phase contrast microscopy leverages differences in the refractive index between specimens and their surrounding medium. It translates phase shifts into amplitude differences through interference with unaltered light. Contemporary phase contrast microscopes achieve this using a condenser annulus in the illumination path and a phase ring in the objective lens, which modifies the light phase. Proper alignment of these components is essential to establish phase contrast conditions (Murphy and Davidson 2012; Maurer et al. 2008).

Despite its advantages, present technology confines phase contrast microscopy to regions near the center of liquid‐filled vessels due to the meniscus effect (Fuchs et al. 2023). This effect, induced by capillary forces, results in an inclined liquid surface that refracts incident light at the liquid‐air interface, causing misalignment between the condenser annulus and the phase ring (Horn and Zantl 2006). Consequently, phase contrast conditions cannot be properly established (Hofmeister et al. 2020). Biological samples, such as cell cultures, demonstrate heterogeneous behavior across the well surface. Cells in the center experience different levels of resource access and competition, influencing their growth (Mansoury et al. 2021). Focusing solely on the center can lead to inaccurate interpretations. However, specimens near the edges of vessels remain unobservable with phase contrast microscopy, a limitation that becomes more significant as vessel size decreases. In typical cell culture vessels, such as MTPs, only a small portion of the surface is observable, particularly in high‐order MTPs. For example, in 24‐well MTPs, the observable area is estimated to be approximately 8.6% when using 10× magnification (Nienhaus et al. 2023). Therefore, it is essential to maximize the observable sample area by developing methods to mitigate or compensate for the meniscus effect. This paper proposes one such method. Additionally, estimates of the phase contrast area are only approximations due to the lack of a standardized method for quantifying this area. Thus, we also introduce a novel approach in this publication (Rubin 1990).

Various approaches have been proposed to mitigate the meniscus effect. These include preventing meniscus formation with specialized cell culture vessels, using uniquely designed MTPs to correct optically, altering the optical path within the microscope, and employing image analysis techniques (Nienhaus et al. 2023). One notable approach is outlined in US patent US 9,069,175 B2 (Douglas 2012). This method replaces the fixed condenser annulus unit with a transmissible liquid crystal display (LCD), allowing flexible adjustment. Building upon this idea, we developed an automated process for acquiring images of an entire well in an MTP. Our primary objective is to implement an easy‐to‐use method that efficiently determines the parameters required for an LCD to display a virtual condenser annulus, compensating for the meniscus effect at each position within an MTP well. This process must be both time‐efficient and scalable for future integration into fully automated sample screening systems.

## 2 Materials and Methods

We constructed a demonstrator utilizing a commercial phase contrast microscope, in which the condenser annulus unit was replaced with a transmissible LCD. A process was developed to scan an entire well by acquiring and stitching multiple images. For each individual image, data from a secondary camera connected to a Bertrand lens was employed to calculate the parameters necessary for displaying a virtual condenser annulus on the LCD. To validate the performance, we devised an algorithm to quantify the phase contrast area within a composite image of the entire well. Consistent with previous publications, we call the method adaptive phase contrast microscopy (Nienhaus et al. 2023; Douglas 2012; Schenk 2016).

### 2.1 Experimental Setup

**FIGURE 1:**
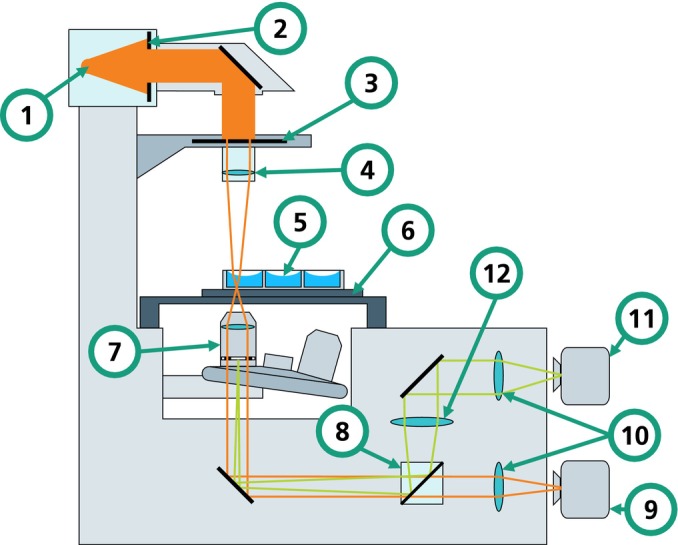
Schematic drawing of the phase contrast microscope used for all experiments. (1) Light source, (2) aperture, (3) LCD condenser annulus, (4) condenser lens, (5) MTP, (6) microscope stage, (7) objective lens with phase ring, (8) rotatable mirror, (9) primary camera, (10) tube lenses, (11) secondary camera, and (12) Bertrand lens.

The experimental setup, depicted in Figure 1, is based on an Eclipse Ti2‐E inverted microscope (Nikon, Japan). The original condenser unit was replaced with a custom adapter housing a transmissible LCD (VS055QUM‐NH0‐6KP0, BOE, China). This monochrome LCD, which does not feature a backlight, was specifically selected due to its lack of color filters that could further attenuate light, beyond the unavoidable attenuation due to the LCD's working principle (Yang and Wu 2015).

The LCD connects to a Raspberry Pi 4B via HDMI, which then links to a Windows PC through Ethernet. During image acquisition, the main program running on the Windows PC calculates the condenser annulus parameters and sends them to the Raspberry Pi. The Raspberry Pi renders a ring as a black‐and‐white matrix on the LCD, emulating a condenser annulus.

A condenser lens (ELWD, Nikon, Japan) is affixed to the LCD adapter. The light source (LE CG P3AQ, Osram, Germany) emits green light with a peak wavelength of 520 nm, closely matching the design wavelength of the phase ring. Its high luminous flux, reaching up to 18,000 lm, ensures adequate image brightness despite LCD attenuation. Experiments use 24‐well MTPs filled with fixed mesenchymal stem cells (MSCs) (Gnecchi and Melo 2009). These plates are positioned on a piezo stage (SPS‐D90500, nanoFactur, Germany) for rapid focusing, mounted on a microscope stage (SCANplus IM, Märzhäuser Wetzlar, Germany) for XY movements. An objective lens with a magnification of 10× and a numerical aperture of 0.3 is consistently used (CFI Plan Fluor DL 10XF, Nikon, Japan). The light path is aligned to ensure Köhler illumination. Images of the samples are captured by the monochrome main camera (VC‐5MX2‐M/C289, Vieworks, Republic of Korea). To examine the overlap of the phase ring and condenser annulus, a secondary camera of the same model is mounted on a side port, facilitating imaging through the Bertrand lens of the microscope. A rotatable mirror in the microscope directs the light beam to either camera.

### 2.2 Image Acquisition Process

**FIGURE 2:**
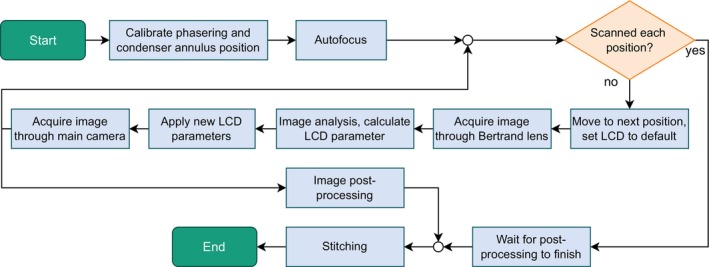
Image acquisition process for the adaptive phase contrast microscopy.

The objective is to acquire a high‐resolution phase contrast image of an entire well. Because of the microscope's limited field of view, the well is divided into multiple images, which are stitched together later. Figure 2 schematically illustrates this process.

In the initial step, an algorithm calculates the coordinates for all image positions based on the MTP well dimensions so that the whole area is covered with small overlaps between images. An optional calibration ensures proper LCD parameter calculation. During calibration, the microscope stage aligns the well center with the optical center to minimize the meniscus effect. The LCD is set to a white screen, creating brightfield conditions. The secondary camera, through the Bertrand lens, captures an image of the phase ring, which appears black against the white background. An image analysis algorithm, explained in the following section, calculates the phase ring center, serving as a reference for subsequent images. Next, the LCD displays a condenser annulus at a default position. Another image is captured through the Bertrand lens, and image analysis determines a new default position.

The autofocus scan determines the z‐position of the sample throughout the well, ensuring focused images across the entire area. Autofocus is performed by image stacking at four equally spaced well positions. A sharpness algorithm calculates the optimal focus at each location. Linear interpolation and extrapolation determine focal positions for all images (Narrog et al. 2023).

Subsequently, the actual acquisition process commences. The following steps are carried out for each individual image that contributes to the complete visualization of the well. First, the microscope stage moves the sample to the next position, and the LCD is set to display the default condenser annulus. A resting period of 200 ms is then applied to allow the liquid to settle after being accelerated and decelerated during movement. An image is then captured by the secondary camera, and an image analysis algorithm (described in the next paragraph) calculates the required position of the condenser annulus on the LCD. These parameters are transmitted to the Raspberry Pi, which adjusts the LCD to display an annulus based on the computed values. Next, the microscope switches the rotatable mirror, allowing the main camera to capture an image, which is saved as part of the final composite image. Parallel to the acquisition process, post‐processing techniques, namely histogram adjustment and shading correction, are applied to each image after it is captured. Upon completion of image acquisition and post‐processing, a stitching algorithm combines the individual images into a single, cohesive large image.

### 2.3 Derivation of LCD Condenser Annulus Parameters

The LCD parameters are determined through a two‐step process: first, image analysis of Bertrand lens images is conducted with the LCD condenser annulus in its default position to calculate the displacement of the condenser annulus center; second, new LCD parameters are derived using linear regression.

#### 2.3.1 Image Analysis Algorithm

The goal of the image analysis algorithm is to determine the offset between the condenser annulus and the phase ring, represented as $\Delta \vec{d}$. The centers of these rings are detected using a series of classical computer vision techniques. Distinct parameter sets were used for the condenser annulus and the phase ring to address the different lighting conditions and the less consistent shape observed in the condenser annulus images.

In a first step, the input images are scaled down to 20% to reduce computation time. Contrast limited adaptive histogram equalization (CLAHE) is then applied to enhance the brightness in areas of the image darkened by the phase ring (Pizer et al. 1987). Following this, a binary threshold is applied to segment a continuous ring. The histogram equalization and thresholding parameters are fine‐tuned to achieve this segmentation. The contour‐detection algorithm by Suzuki and be (1985) is used to extract the outer edge of the ring. To eliminate false positives caused by reflections, constraints on size and roundness, derived from the contours, are applied. Roundness o is calculated using Formula (1):

(1) $$o=\frac{4\mathrm{\pi A}}{P^{2}}$$

where A stands for the area and P for the perimeter of the contour.

To address potential irregularities in the contour due to dimming from the phase ring, a convex hull is computed. An ellipse is then fitted to the outer contour using an approximate mean square algorithm (Taubin 1991). The ellipse is preferred over a circle due to the distortion of the condenser annulus near the well's edge, which is a result of the meniscus's concave shape.

This algorithm can be computed in less than 10 ms on the used computer, thereby making it suitable for a high‐speed process.

#### 2.3.2 Linear Regression

Following the image analysis, the LCD parameters must be derived from the calculated center deviations. Due to the linear characteristics of the optical components between the condenser annulus and the phase ring, there is a linear relationship between the horizontal shift of the condenser annulus and the observed displacement on the phase ring plane. Thus, the relationship between the position of the condenser annulus displayed on the LCD, denoted as $\vec{p}$ (in mm), and the observed displacement through the Bertrand lens, $\Delta \vec{d}$ (in pixels), can be described by a linear dependency, as expressed in Formula (2):

(2) $$\vec{p}=k\Delta \vec{d}+{\vec{p}}_{0}$$

where ${\vec{p}}_{0}$ represents the default position where condenser annulus and phase ring are perfectly aligned in the absence of a meniscus, and k is the scaling factor to be determined. The scaling factor k was empirically determined through experiments, as analytical calculation is challenging due to various system tolerances and the proprietary nature of the lens design.

Experiments were conducted to find pairs of $\vec{p}$ and $\Delta \vec{d}$ for the calculation of k. To achieve this, several positions were explored in multiple liquid‐filled MTPs. At each position, an image was initially captured with the LCD condenser annulus set to ${\vec{p}}_{0}$. The ring displacement $\Delta \vec{d}$ was calculated using the image analysis algorithm. Subsequently, the condenser annulus was manually adjusted until it was concentric with the phase ring, and this position was recorded as $\vec{p}$.

**FIGURE 3:**
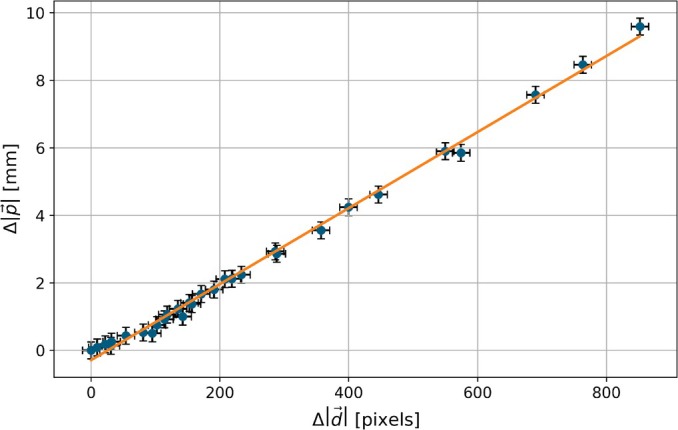
Regression line for calculating the scaling factor k. The *x*‐axis shows the condenser annulus displacement, $\Delta \left|\vec{d}\right|$, as observed through the Bertrand lens with default annulus parameters. The *y*‐axis displays the necessary shift on the LCD, $\Delta \left|\vec{p}\right|$, to compensate for this displacement. Error bars on the *x*‐axis represent the 75 percentile of image analysis error (see section). *Y*‐axis error bars indicate an estimated manual error of 0.25 mm, based on experiments in Section 2.

Since k is identical in both the x and y directions, the resulting absolute values for $\Delta \left|\vec{d}\right|$ and $\Delta \left|\vec{p}\right|=\left|\vec{p}-{\vec{p}}_{0}\right|$ could be used to be plotted on a graph, and a regression line was calculated, as illustrated in Figure 3. The scaling factor k was determined to be 0.0113 mm/px. The minor deviations from the regression line and absence of outliers, underlined by the error bars, confirm that a linear relationship is applicable.

### 2.4 Algorithm to Quantify Phase Contrast Conditions

To quantify the increase in phase contrast area from the adaptive phase contrast microscopy approach, a measurable metric for the phase contrast area is essential. The objective was to develop an algorithm that takes a phase contrast image of an entire well and outputs the relative phase contrast area.

#### 2.4.1 Characteristic Parameters for Phase Contrast Conditions

**FIGURE 4:**
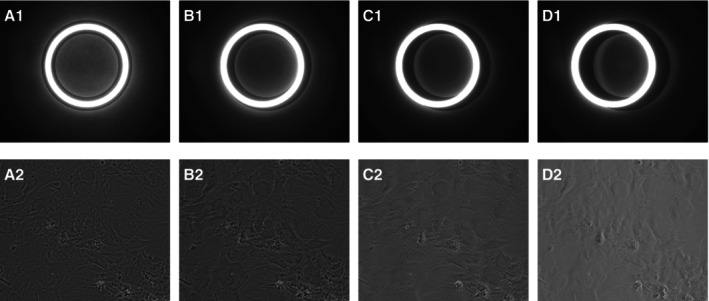
Superposition of condenser annulus and phase ring viewed through a Bertrand lens (A1—D1) and their corresponding phase contrast images of MSCs as viewed through the main camera (A2—D2). The condenser annulus position on the LCD is shifted by 0.0, 0.5, 1.0, and 1.5 mm from the center for A–D, respectively.

Prior to algorithm development, we analyzed phase contrast conditions using real images. Images of MSCs were captured under both phase contrast and non‐phase contrast conditions in a liquid‐filled well of a 24‐well MTP. The decision to use MSCs was solely to obtain a sample with sufficient contrast; other biological samples could also be suitable. Three distinct positions were examined: Position 1 (center), Position 2 (2 mm from the center), and Position 3 (4 mm from the center). At each position, the virtual condenser annulus on the LCD was adjusted to align concentrically with the phase ring as default. One image was captured from each camera. The virtual condenser annulus was then shifted by 0.25 mm on the LCD, and the next set of images was acquired, continuing this process until the phase ring and condenser annulus no longer overlapped. The results for Position 1 are illustrated in Figure 4, showcasing every second set of images. Subfigure A1 shows the complete alignment and overlap of the rings, producing the phase contrast image in A2. In subfigure B1, the rings are no longer concentric but still overlap, resulting in no significant change in B2. In contrast, image sets C and D demonstrate lower contrast and in average brighter images.

Using these image sets, we compared the characteristics of phase contrast images with those lacking complete phase contrast. Phase contrast conditions are identified by enhanced contrast features and a darker background, distinguishing them from brightfield conditions (Murphy and Davidson 2012). As measuring two metrics simultaneously can lead to ambiguity, it is essential to identify a single metric that reliably predicts phase contrast conditions in an image.

**FIGURE 5:**
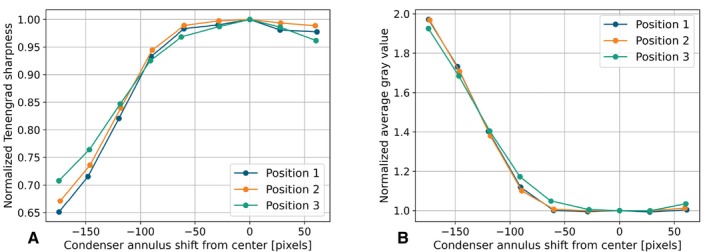
Relative sharpness and average gray values plotted against the deviation of the condenser annulus position from the center in pixels, as observed through the secondary camera. Both values are normalized to the measurements at Position 1, with zero deviation from the center. (A) Relative sharpness. (B) Average gray value for each phase contrast image.

We quantified contrast and background brightness for each image, as shown in Figure 5. Contrast is represented as sharpness, calculated using the Tenengrad method (Zhu et al. 2023) (subfigure 5A). Background darkness was assessed by computing the average intensity across the entire image (subfigure 5B).

We plotted the resulting values against the phase ring's distance from its initial position. To compare across all three positions, sharpness and brightness values were normalized to those at the central condenser annulus position. Figure 5 shows that sharpness peaks when the condenser annulus and phase ring overlap. As the overlap decreases, sharpness diminishes. Similarly, image brightness is minimized when the rings overlap but increases sharply once they no longer do. Consequently, the brightness and sharpness curves show a strong negative correlation, with a Pearson correlation coefficient of −0.994 (Benesty et al. 2009).

As a result, both metrics—image contrast and background brightness—can independently determine phase contrast conditions, given a reference point known to exhibit such conditions, like the center of a well. However, image contrast can be influenced by multiple factors, including focus sharpness and specimen characteristics. High MSC density areas may appear sharper despite lower contrast per feature.

Consequently, background brightness is a more robust metric, assuming constant lighting conditions. Therefore, we have chosen background brightness as the independent metric to characterize phase contrast conditions.

#### 2.4.2 Algorithm

**FIGURE 6:**
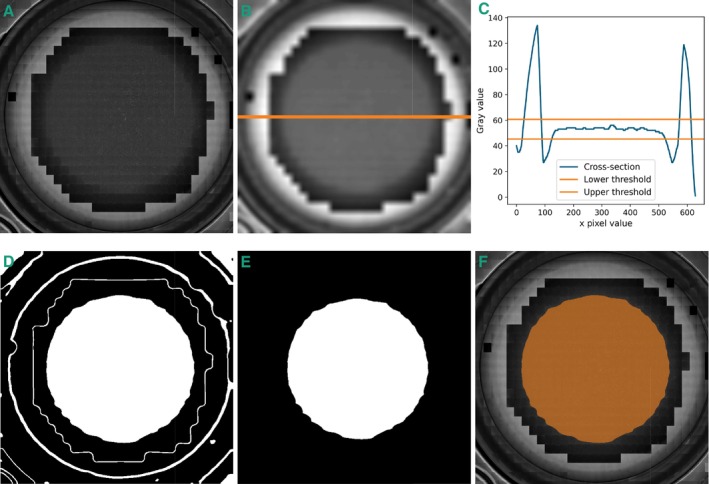
Image analysis algorithm to determine the phase contrast area. (A) Original image of a well acquired with adaptive phase contrast microscopy. (B) Blurred and compressed image. The orange line represents the position of the central cross‐section. (C) Gray values at the cross section with threshold. (D) Binarization of image B. White areas lie within the threshold. (E) Image after erosion and selecting the central white patch. (F) Calculated phase contrast area overlaid on original image.

We developed an algorithm based on background brightness to calculate the phase contrast area within a full‐well image. The steps are illustrated in Figure 6. Because of its composition of hundreds of individual microscope images, the original file size exceeds 1 GB, so the initial step scales down the image. An example adaptive phase contrast image is shown in subfigure 6A.

Next, we apply a Gaussian filter to blur the image, reducing detail and enhancing background color dominance in each local area (subfigure 6B). The following step determines upper and lower thresholds for the background color to qualify as phase contrast. Assuming the well is photographed centrally, the center of the meniscus is at the image's center, where phase contrast conditions should be present. The gray value at the center is therefore considered the base gray value for phase contrast. Since the gray values vary within the phase contrast area, we postulate that gray values indicative of phase contrast must be within a lower and upper threshold. We opted for a relative threshold because a fixed pixel value threshold is not robust due to fluctuating lighting conditions across multiple composed images. This threshold is determined by identifying bright values that represent brightfield conditions. We achieve this by drawing a cross‐section through the image center and calculating the mean of the highest values. We specify that the threshold value should lie between the brightest and the darkest gray value, with the threshold being closer to the dark value. Consequently, we defined the thresholds $t_{\text{upper}}$ and $t_{\text{lower}}$ as 15% intervals between the gray value at the center $g_{\mathrm{ph}}$ and the brightfield gray value $g_{\mathrm{br}}$, expressed as:

(3) $$\left(\begin{array}{c} t_{\text{upper}}\\ t_{\text{lower}} \end{array}\right)=g_{\mathrm{ph}}\pm 0.15\left(g_{\mathrm{br}}-g_{\mathrm{ph}}\right)$$

A cross‐sectional view of gray values through the image, including both thresholds, is shown in subfigure 6C.

After establishing the thresholds, binarization identifies all pixel values within the upper and lower boundaries, depicted in subfigure 6D. However, this step may include regions outside the central area with similar gray values. To isolate the central region, erosion is applied to sever connections to peripheral regions. Next, we retain only the most central patch, discarding all other areas. This results in a single continuous region surrounding the center, illustrated in subfigure 6E. The final step involves dilation to reconstruct the original patch around the center.

The resulting phase contrast area, overlaid on the resized original image, is depicted in subfigure 6F. To calculate the proportion of the phase contrast area relative to the total well area, we divide the size of the phase contrast area in pixels by the total well area, modeled as a circle of known diameter.

## 3 Results

The results are organized into two main sections. First, we present a benchmarking test for the image analysis algorithm. Second, we discuss the restoration of phase contrast conditions using adaptive phase contrast microscopy.

### 3.1 Image Analysis

We conducted a comprehensive study to assess the performance of the image analysis algorithm. A total of 500 images of the condenser annulus were captured at various positions. The ring centers in these images were manually labeled as reference using a tool that enables users to draw rings on the images and record the center points in a JSON file. Subsequently, the algorithm predicted the ring centers, and the discrepancies between the computed and labeled centers were examined.

**FIGURE 7:**
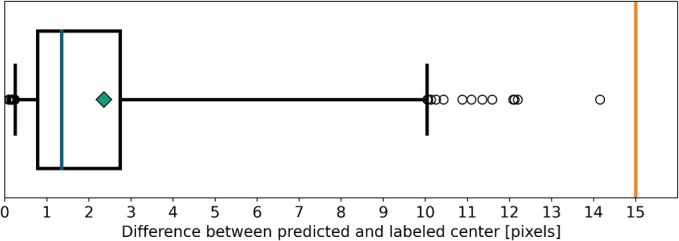
Error distribution between predicted and labeled ring centers. Since all images are scaled to 20% size, the real center deviations are five times larger. Median = 1.4 pixels, mean = 2.4 pixels, standard deviation = 2.6 pixels, whiskers at 2.5th and 97.5th percentile. Threshold for acceptable images at 15.0 pixels.

As previously established, the centers of the condenser annulus and the phase ring do not need to align perfectly to produce phase contrast conditions. To establish an acceptable error margin, we analyzed images A1 and B1 from Figure 4. The centers of these images were labeled and their distances calculated. The shift between the two rings was determined to be 15.0 pixels after scaling down the images, which we set as the acceptable margin for phase contrast conditions. The error distribution from the study is illustrated in the boxplot shown in Figure 7. For every single image, the error is well below the acceptable threshold, demonstrating the algorithm's high reliability. The small residual differences between the predicted and labeled centers can partly be attributed to the inaccuracy of manual labeling, which may introduce errors of several pixels so that the actual error of the algorithm is even smaller.

### 3.2 Adaptive Phase Contrast Process

To evaluate the whole process, a series of experiments was conducted following the established methodology, using the described demonstrator. Fixed MSCs in a 24‐well MTP serve as the sample. The MSCs do not alter the phase contrast conditions; they are employed solely as a contrasting example and due to their adherent properties. We capture images from three out of the 24 wells within the MTP for analysis. To validate the method we acquired images of these same wells with the same setup except for using the default condenser unit, which replaces the LCD adapter.

**FIGURE 8:**
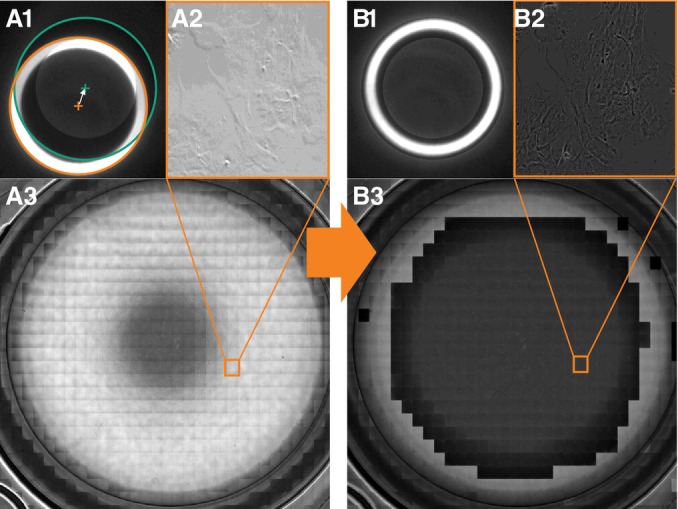
The Bertrand lens image (A1) shows a condenser annulus displacement, resulting in an image without phase contrast conditions (A2). Due to image analysis and LCD condenser annulus shifting, the condenser annulus aligns with the phase ring in (B1), resulting in the phase contrast image (B2).

**FIGURE 9:**
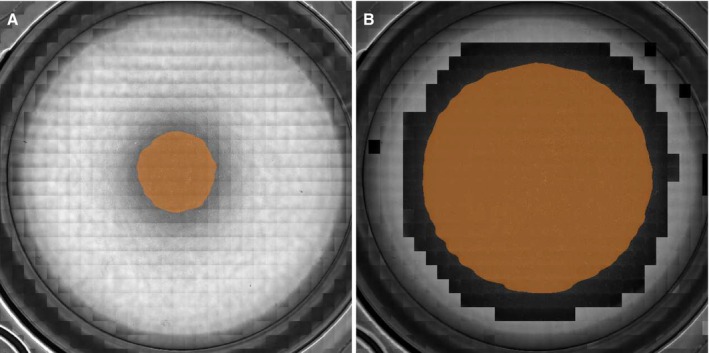
Compound images of a single MTP24 well. (A) Regular phase contrast microscopy (B) adaptive phase contrast microscopy. Phase contrast area is marked in orange.

The final images are stitched together out of 864 images and the acquisition time is approximately 40 min. Figure 8 shows results for conventional and adaptive phase contrast microscopy. Following image acquisition, we applied the phase contrast area quantification algorithm across all images. The outcomes are illustrated in Figure 9.

Measurements obtained from the three wells yield a consistent increase in phase contrast area, as illustrated in Table 1. The image analysis and regression of the annulus parameters were executed flawlessly within the relevant area, indicated by a complete overlap between the condenser annulus and the phase ring. This is visually apparent in the final images, which display a continuous region with a darker background. Moving away from the center of the well, the brightness remains nearly constant over a significant portion, before ultimately decreasing. The slow decrease is followed by a rapid increase in brightness, indicating the start of the brightfield area.

**TABLE 1:** The proportion of the well surface area where successful phase contrast imaging was obtained.

Share of phase contrast area in well	Well 1	Well 2	Well 3	Mean	Std.
Conventional condenser annulus	5.0%	5.1%	5.0%	5.0%	0.1%
LCD condenser annulus	42.7%	42.8%	40.3%	41.9%	1.2%
Ratio	8.5	8.3	8.1	8.3	0.1

*Note:* The results of Well 2 correspond to the images in Figures 8 and 9.

With the conventional phase contrast method, the calculated phase contrast area averages around 5.0%. In contrast, the adaptive method yields an average phase contrast area of 41.9%, resulting in an 8.3‐fold increase. In all evaluated images, the phase contrast area is very close to a circular shape, reflecting the rotational symmetry of the wells.

## 4 Discussion

Our findings reveal a significant increase of over eight‐fold in the observable area in well plates using phase contrast microscopy. This increase will allow scientists to conduct comprehensive analyses rather than relying on random sampling of biological samples, facilitating large‐scale specimen examination, which is particularly beneficial for high‐throughput screening (Boutros, Heigwer, and Laufer 2015). Although the precise phase contrast area depends on the threshold value in Equation (3), the overall increase is substantial and consistent. Table 1 illustrates minimal variations across all three wells.

A notable observation is the background around the phase contrast area; it appears brighter with regular phase contrast microscopy and darker with adaptive phase contrast microscopy. This phenomenon can be explained by two key factors. Firstly, an increased meniscus angle leads to greater light reflection rather than refraction, as described by the Fresnel equation (James and Agarwal 1996). Second, the condenser lens aperture is insufficiently large, causing only a portion of the condenser annulus to be visible on the meniscus surface, resulting in a sickle‐shaped illumination rather than an annulus.

The image analysis algorithm accurately identified all condenser annuli and calculated the appropriate LCD values, ensuring effective meniscus compensation in the central area. Image analysis remains effective as long as the condenser annulus is positioned to appear as a complete ring through the Bertrand lens. In cases where the ring is not fully visible, the LCD displays the default annulus, resulting in brightfield conditions. Occasionally, the algorithm erroneously interprets certain features as rings, leading to incorrect LCD values. This can be observed as dark patches located at the outer edge of the well. Nonetheless, these limitations are inconsequential since phase contrast conditions cannot be achieved in these peripheral areas. The algorithm employs a brightness threshold to detect ring shapes, which may require adjustment under varying lighting conditions.

The novel algorithm developed to calculate the phase contrast area allows for quantitative comparison between methods and exhibits less variation than manually labeled images. This accounts for the discrepancy in phase contrast area measurements between this study and our previous work. In this study, we measured a phase contrast area of 5.0% for conventional phase contrast microscopy in 24‐well MTPs, whereas our previous study reported 8.6%, based on expert opinion (Nienhaus et al. 2023).

The proposed approach is simple to implement and effectively compensates for the meniscus effect. In contrast to Hofmeister et al. (2020), who used a digital micromirror device (DMD) to replace the condenser annulus, our method does not require a complete overhaul of the illumination path, simplifying integration with existing phase contrast microscopes. While Hofmeister et al. claim to achieve phase contrast near the well edge in a 24‐well plate, which likely exceeds the 41.9% phase contrast area of our method, their results need validation under identical conditions for a fair comparison.

The acquisition process proved to be time‐consuming, taking approximately 40 min for a single well. This was mainly due to the careful movement of the sample and the necessary waiting time to prevent the liquid from sloshing, which could unpredictably alter the shape of the meniscus surface. Future research could explore methods to accelerate the acquisition process.

A key advantage of the presented approach is its cost‐effectiveness. The required hardware, consisting of an LCD and controller, housing, and a secondary camera, can be assembled for less than 1000 USD, with no need for other modifications to the microscope. This is significantly cheaper than a DMD‐based setup with a different illumination path (Hofmeister et al. 2020). Moreover, the proposed method is more economical for repeated use compared to special MTPs designed to eliminate or correct the meniscus effect, as these are single‐use products (Klöcking, Wönne, and Wutzler 1999).

## 5 Conclusion and Outlook

We developed a method to compensate for the meniscus effect in phase contrast microscopes using an LCD instead of a conventional condenser annulus. The primary focus was on a procedure to derive parameters for the LCD and creating an algorithm to quantify phase contrast conditions in microtiter plates. The implementation involves using a secondary camera to acquire images through a Bertrand lens, combined with image analysis to determine the LCD parameters, offering a robust and straightforward procedure. The more than eight‐fold increase in phase contrast area enables operators to observe larger samples and detect features closer to the well edge.

The simplicity of the setup and algorithm paves the way for future commercialization. It is conceivable that microscope manufacturers could offer an adaptive phase contrast module as an add‐on to their microscopes. Moreover, an improved process to enable faster image acquisition would further increase the appeal of the technology. The absence of moving parts enhances the robustness of the hardware and allows for high‐speed imaging, comparable to the work of Schenk et al. (2016). Future studies could explore the feasibility of achieving a phase contrast area covering 100% of the well surface, particularly for higher format MTPs. Additionally, the development of customized LCDs that minimize light obstruction and offer faster frame switching times could enhance both image quality and acquisition speed.

## Author Contributions

**Florian Nienhaus:** conceptualization, investigation, funding acquisition, writing – original draft, methodology, validation, visualization, writing – review and editing, software, formal analysis. **Finn Burkhardt:** writing – original draft, software, validation, formal analysis. **Niels König:** conceptualization, supervision, funding acquisition, project administration, data curation. **Robert H. Schmitt:** project administration, supervision, resources.

## Supporting information


**Data S1.** Results conventional low compression.

**Data S2.** Results LCD low compression.

## Data Availability

The data that supports the findings of this study are available in the Supporting Information of this article.
