# Supplementary figures and images for: Compensating the Meniscus Effect in Phase Contrast Microscopy Using an LCD for Adaptive Condenser Annulus Shifting
Source: Microsc Res Tech. 2025 Jan 17;88(5):1534–43. doi: 10.1002/jemt.24808 (PMC11972447; doi:10.1002/jemt.24808)

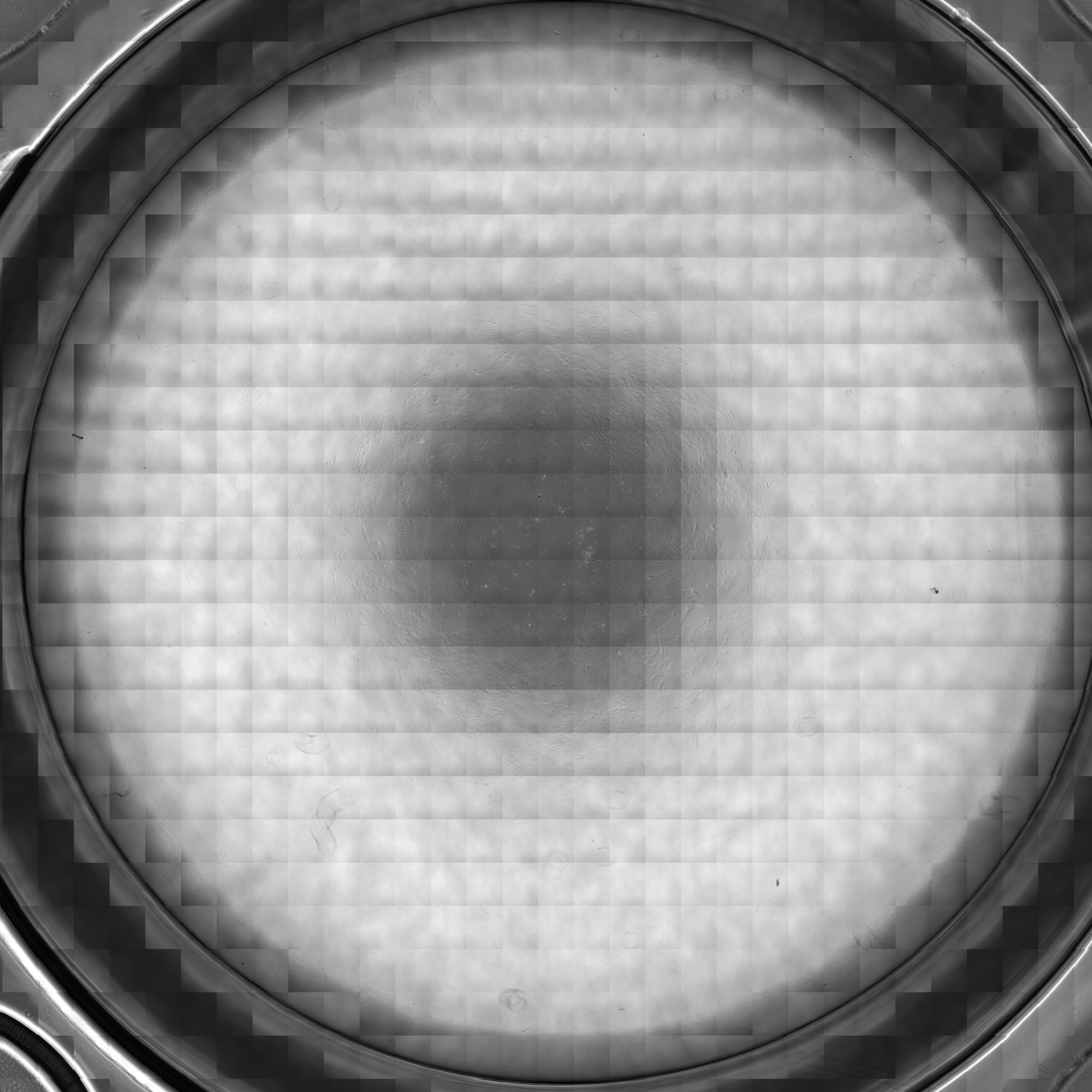

Supplement: Supplementary file 1 — Data S1. Results conventional low compression. [file JEMT-88-1534-s001.png]

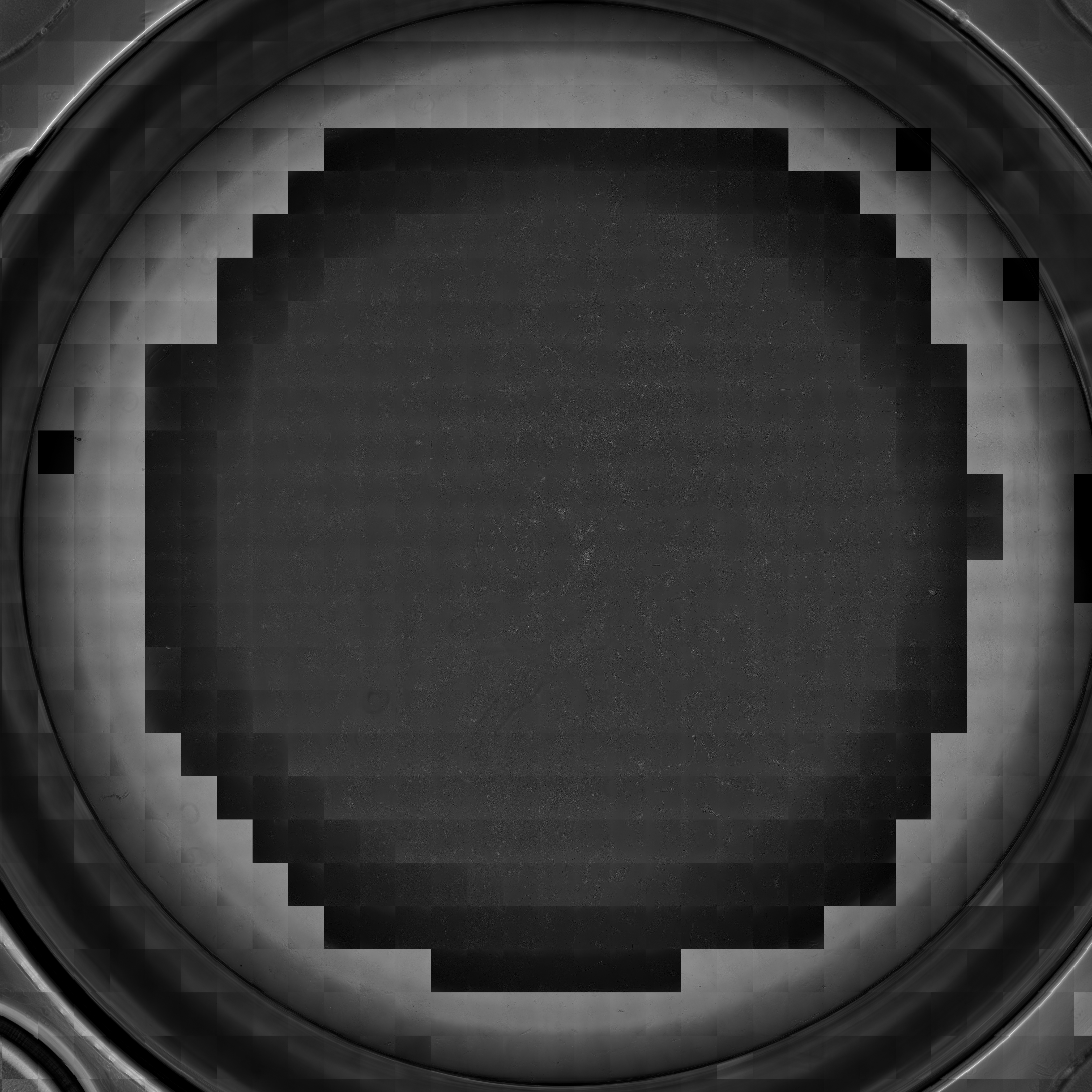

Supplement: Supplementary file 2 — Data S2. Results LCD low compression. [file JEMT-88-1534-s002.png]
